# Development of a new poly-ε-caprolactone with low melting point for creating a thermoset mask used in radiation therapy

**DOI:** 10.1038/s41598-021-00005-2

**Published:** 2021-10-14

**Authors:** Takahiro Aoyama, Koichiro Uto, Hidetoshi Shimizu, Mitsuhiro Ebara, Tomoki Kitagawa, Hiroyuki Tachibana, Kojiro Suzuki, Takeshi Kodaira

**Affiliations:** 1 Department of Radiation Oncology, Aichi Cancer Center, 1-1 Kanokoden, Chikusa-Ku, Nagoya, Aichi 464-8681 Japan; 2 Graduate School of Medicine, Aichi Medical University, 1-1 Yazako-karimata, Nagakute, Aichi 480-1195 Japan; 3 Research Center for Functional Materials, National Institute for Materials Science, 1-1 Namiki, Tsukuba, Ibaraki 305-0044 Japan; 4 Department of Radiology, Aichi Medical University, 1-1 Yazako-karimata, Nagakute, Aichi 480-1195 Japan

**Keywords:** Chemical engineering, Design, synthesis and processing, Head and neck cancer

## Abstract

This study aimed to develop a poly-ε-caprolactone (PCL) material that has a low melting point while maintaining the deformation ability. The new PCL (abbreviated as 4b45/2b20) was fabricated by mixing two types of PCL with different molecular weights, numbers of branches, and physical properties. To investigate the melting point, crystallization temperature, elastic modulus, and elongation at break for 4b45/2b20 and three commercially available masks, differential scanning calorimetry and tensile tests were performed. The melting point of 4b45/2b20 was 46.0 °C, and that of the commercially available masks was approximately 56.0 °C (55.7 °C–56.5 °C). The elastic modulus at 60 °C of 4b45/2b20 was significantly lower than the commercially available masks (1.1 ± 0.3 MPa and 46.3 ± 5.4 MPa, *p* = 0.0357). In addition, the elongation at break of 4b45/2b20 were significantly larger than the commercially available masks (275.2 ± 25.0% and 216.0 ± 15.2%, *p* = 0.0347). The crystallization temperature of 4b45/2b20 (22.1 °C) was clinically acceptable and no significant difference was found in the elastic modulus at 23 °C (253.7 ± 24.3 MPa and 282.0 ± 44.3 MPa, *p* = 0.4). As a shape memory-based thermoset material, 4b45/2b20 has a low melting point and large deformation ability. In addition, the crystallization temperature and strength are within the clinically acceptable standards. Because masks made using the new PCL material are formed with less pressure on the face than commercially available masks, it is a promising material for making a radiotherapy mask that can reduce the burden on patients.

## Introduction

In radiotherapy for head and neck cancer, a patient-specific thermoplastic mask is created to improve the patient’s positional accuracy because head and neck cancer is often in proximity to critical organs such as the brain stem, spinal cord, and optic nerves^1–8^. Poly-ε-caprolactone (PCL), which has thermoset properties, is a widely used material in commercially available masks^9^. The melting point of PCL is generally around 55 °C–60 °C^10,11^. To create the mask, it is necessary to heat PCL above its melting point, which creates the risk of burns, a physical burden due to the use of sedatives for pediatric patients, and a mental burden due to pressing a high-temperature material against the patient’s face^3,12^. Thus, the current commercially available masks are of great disadvantage to the patients.

To lower the melting point of PCL, the molecular weight of the material should be decreased^13,14^. However, when the melting point of PCL is lowered, its deformation ability reduces^15^. In clinical practice, because the mask is molded by pressing the heated PCL against the patient's face, the pressure applied when creating the mask is increased, or a mask with sufficient accuracy cannot be created when the deformation ability of the PCL is reduced. In recent years, to eliminate the burden on patients caused by heat against their face, many reports have detailed the efficacy of patient-specific masks using three-dimensional print technology (3D printed mask)^16–23^. However, 3D-printed masks do not have widespread clinical use yet owing to many problems that remain to be solved, such as production technology, cost, time limits, and dose verification^20,21^. Thermoplastic polyurethane, which is used in body immobilization systems^24^, is inappropriate for use as a mask because hydrolysis occurs and the stability of the mask decreases when it is immersed in high-temperature water^25,26^. In contrast, PCL is inexpensive, easy to form, and has a long history of being used as a mask for radiotherapy; moreover, the performance of the immobilization system is guaranteed^1–6^. In addition, the use of PCL has been approved by the United States Food and Drug Administration and can be safely used for radiotherapy. Therefore, a mask made of PCL that has a lower melting point while maintaining the deformation ability would be useful; however, to date, no such product exists.

Uto et al. proposed a method for lowering the melting point by mixing two types of PCL with different molecular weights, numbers of branches, and physical properties^27^. According to this method, by changing the mixing ratio, a PCL that has an arbitrary melting point between the melting points of other PCLs can be produced. In addition, the melting point can be lowered while maintaining the deformation ability by combining a PCL that has a low melting point with one that has a large deformability. To investigate this possibility, this study aimed to develop a PCL that has a low melting point while maintaining the deformation ability to reduce the burden on the patient.

## Methods

### Development of the cross-linked PCL

#### Synthesis of branched PCL macromonomer

**Figure 1:**
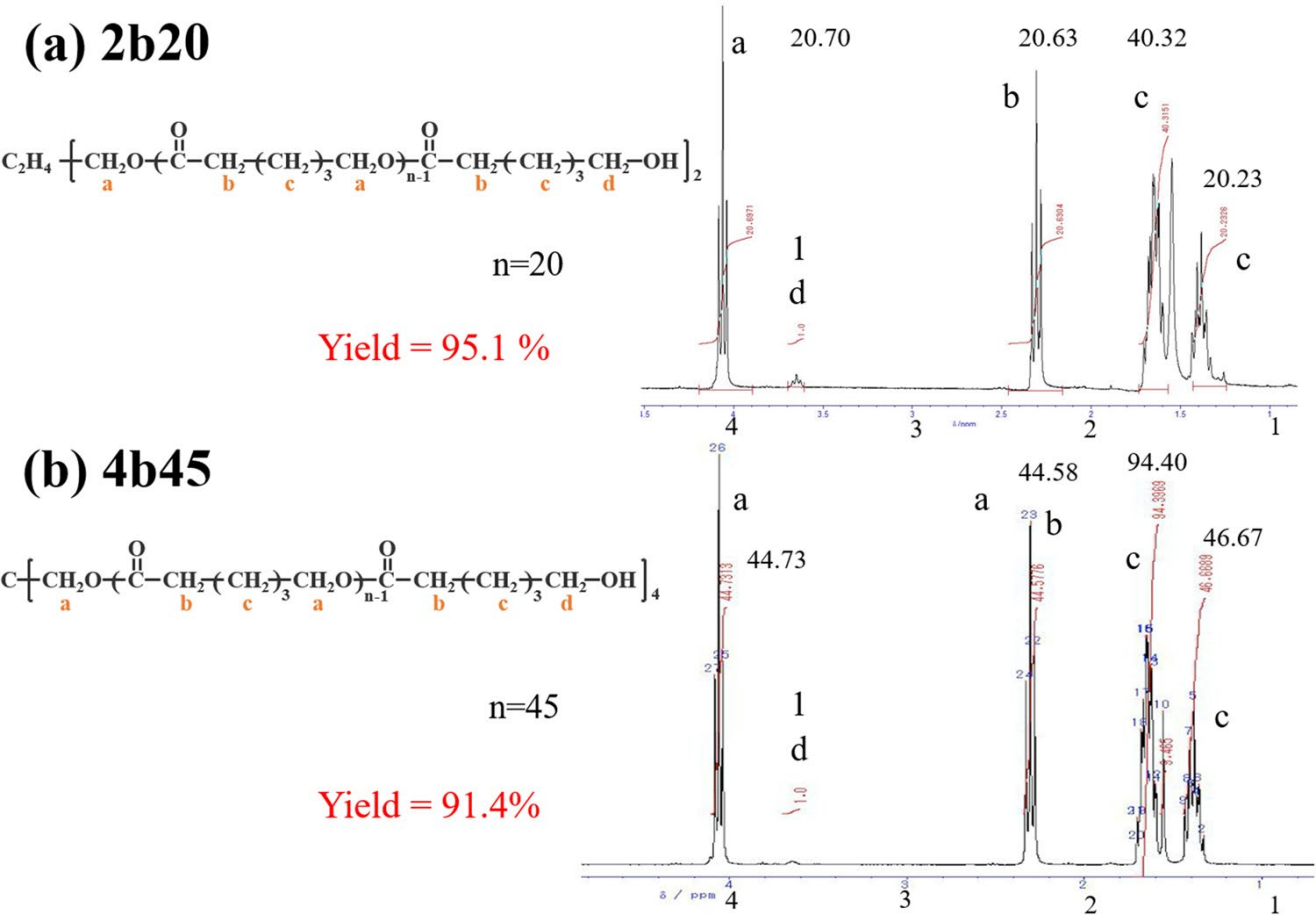
^1^H NMR spectra of linear **(a)** and tetra-branched **(b)** PCL.

**Figure 2:**
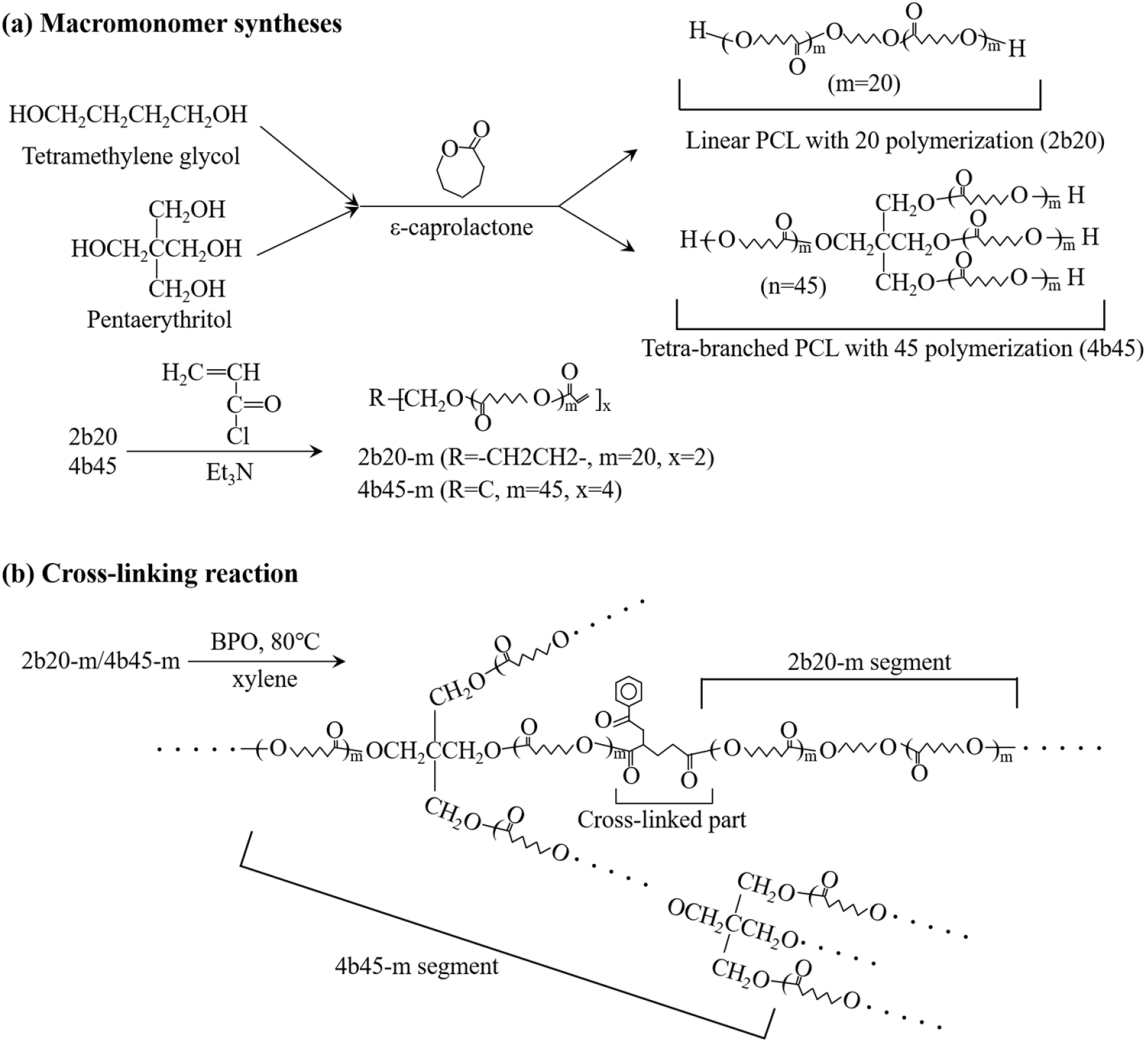
Schematic illustrations of **(a)** macromonomer synthesis and **(b)** cross-linking reaction.

PCL was prepared by cross-linking tetra-branched PCL with acrylate end groups in the presence of linear telechelic PCL with acrylate end groups according to a previously reported protocol^28–30^. Briefly, linear and tetra-branched PCL were synthesized by ring-opening polymerization of ε-caprolactone (Tokyo Chemical Industry [TCI] Co., Tokyo, Japan) that was initiated with tetramethylene glycol (Wako Pure Chemical Industries, Osaka, Japan) and pentaerythritol (TCI) as initiators, respectively. Then, acryloyl chloride (TCI) was reacted to the hydroxyl end group of the branched chains to obtain the macromonomers. The structures and molecular weights were estimated by proton nuclear magnetic resonance spectroscopy (^1^H NMR) (JEOL, Tokyo, Japan). The spectra of the reaction mixture is shown in Fig. 1 and Supplementary figure S1. The average degrees of polymerization of each branch for linear and tetra-branch PCL estimated by ^1^H NMR were 20 and 45, respectively. The linear PCL with average degrees of polymerization of 20 was abbreviated as 2b20, and the tetra-branched PCL with average degrees of polymerization of 45 was abbreviated as 4b45 (Fig. 2).

#### Fabrication of cross-linked PCL macromonomers

The 2b20 and 4b45 macromonomers were dissolved in 50 wt% in xylene containing 1.5 wt% (against polymer) benzoyl peroxide (Sigma-Aldrich, St. Louis, MO, USA) and were mixed at a ratio of 33.3 wt% and 66.7 wt%. The solution was injected between glass slides with a 0.2-mm thick Teflon spacer. The samples prepared using thermal polymerization at 80 °C for six hours to obtain the cross-linked PCL were washed using acetone (a good solvent) and shrunk using methanol (a poor solvent), and then dried for 12 h at a reduced pressure. From these processes, a mixed cross-linked PCL, abbreviated as 4b45/2b20, was fabricated.

### Physical characterization of the developed PCL

#### Measurement of melting point and crystallization temperature of the PCLs

To investigate the melting points of 2b20, 4b45/2b20, and 4b45, the thermal properties were measured (temperature range: 0–100 °C) by differential scanning calorimetry (DSC) (DSC-6100, SEIKO Instruments, Chiba, Japan) at a heating rate of 5 °C/min. In addition, to investigate the crystallization temperature, DSC was performed at − 10 °C/min. The melting point and crystallization temperatures were defined as the endothermic and exothermic peaks on DSC.

#### Measurement of elastic modulus and elongation at break

**Figure 3:**
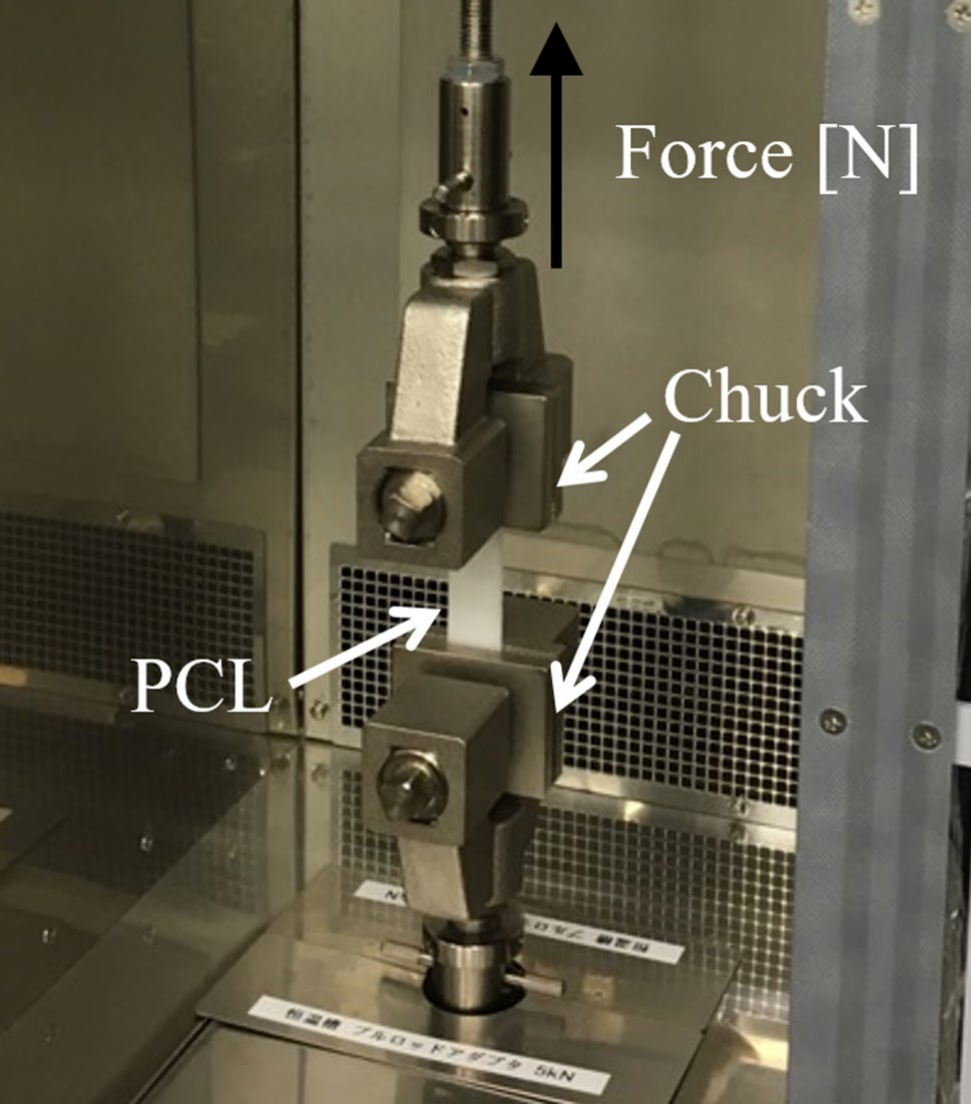
Photographs of the machine for the tensile test. The poly-ε-caprolactone (PCL) is sandwiched between chuck and pulled in the direction of the black arrow at 200 mm/min.

To investigate the elastic modulus of 4b45/2b20 at heating and room temperature, a tensile test (AG-50kNXplus, Shimadzu, Kyoto, Japan) according to the Japanese industrial standards (JIS) K7161-2 was performed at 60 °C and 23 °C. Additionally, the elongation at the break was measured from the tensile test at 60 °C. The sample was attached using probes with a flat screw grip made of cast iron (Fig. 3). As for the 60 °C conditions, the tensile test was started after heating using the test machine for 15 min at 60 °C. The tensile tests were conducted with a loading rate of 200 mm/min. The elastic modulus was determined using the initial slope of the stress–strain curve on the tensile test, and the elongation at break was defined as the materials’ breakage point. Every measurement was repeated three times to confirm the reproducibility.

### Physical characterization of the commercially available masks

#### Measurement of melting point, crystallization temperature, elastic modulus, and elongation at break

To investigate the physical characterization of the three types of commercially available masks Type-S (CIVCO, Iowa, USA), Portrait (Qfix, PA, USA), and Create (Shenzhen Tengfei Yu Technology Co., Shenzhen, China), analysis was performed using DSC (DSC823e, Mettler-Toledo LLC, Tokyo, Japan). Additionally, tensile test using the Type-S mask was conducted at 60 °C and 23 °C. The heating rate of DSC was 5 °C/min and − 10 °C/min, and the loading rate of the tensile test was 200 mm/min. Every measurement was repeated thrice to confirm the reproducibility.

### Statistical analysis

The Mann–Whitney U test was used to compare differences in the elastic modulus and elongation at break between 4b45/2b20 and Type-S. The two-sided p values < 0.05 were significantly different. The statistical analyses were conducted, using EZR version 1.3.6 (Saitama Medical Centre, Jichi Medical University, Saitama, Japan), which is a graphical user interface for R (The R Foundation for Statistical Computing, Vienna, Austria)^31^.

## Results

### Melting point and crystallization temperature

**Figure 4:**
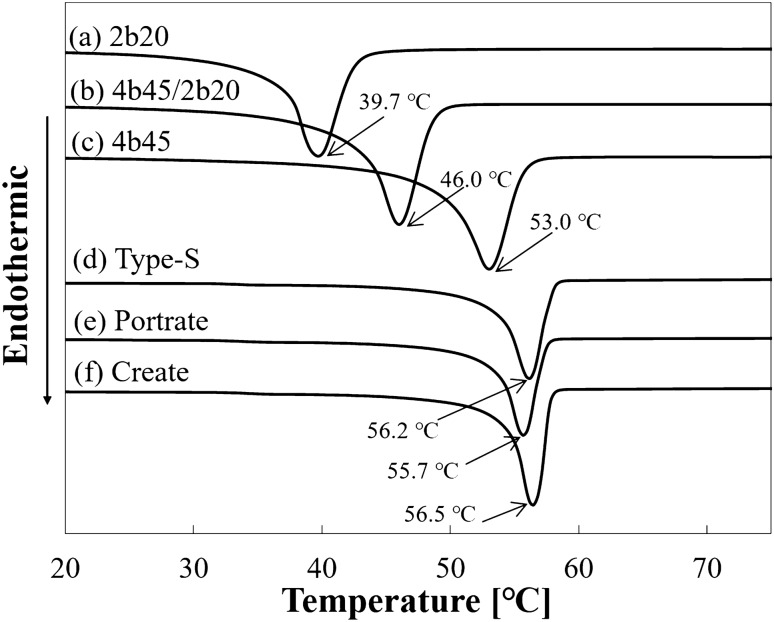
Differential scanning calorimetry charts of the developed poly-ε-caprolactone materials: **(a)** 2b20, **(b)** 4b45/2b20, and **(c)** 4b45, and commercially available masks: **(d)** Type-S, **(e)** Portrait, and **(f)** Create. The melting points measured from endothermic peak are shown.

**Figure 5:**
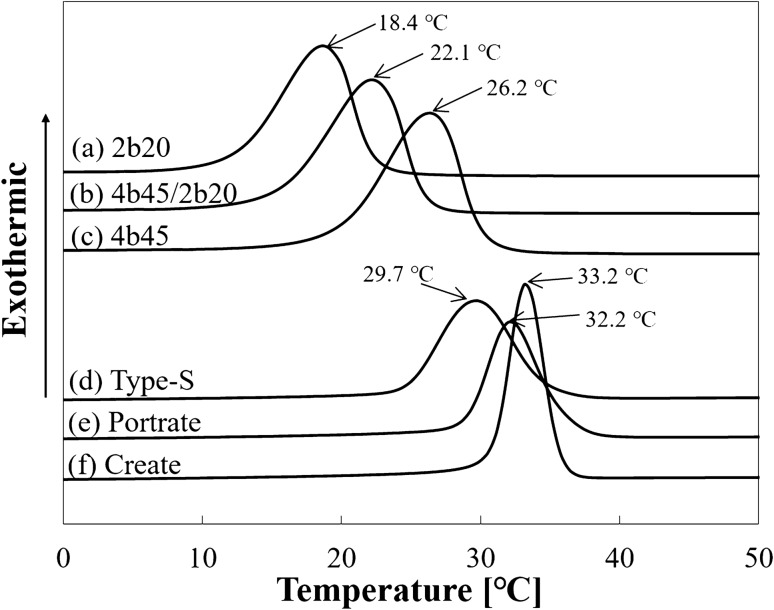
Differential scanning calorimetry charts of the developed poly-ε-caprolactone materials: **(a)** 2b20, **(b)** 4b45/2b20, and **(c)** 4b45, and commercially available masks: **(d)** Type-S, **(e)** Portrait, and **(f)** Create. The crystallization temperatures measured from exothermic peak are shown.

The melting point and crystallization temperatures were evaluated using DSC as presented in “Physical characterization of the developed PCL” and “Physical characterization of the commercially available masks”. The melting points of 2b20, 4b45/2b20, and 4b45 were 39.7 °C, 46.0 °C, and 53.0 °C, respectively, and their crystallization temperatures were 2b20, 4b45/2b20, and 4b45 were 18.4 °C, 22.1 °C, and 26.2 °C, respectively. The melting points of the commercially available masks were 56.2 °C (Type-S), 55.7 °C (Portrait), and 56.5 °C (Create), and their crystallization temperatures were 29.7 °C (Type-S), 32.2 °C (Portrait), and 33.2 °C (Create) (Figs. 4 and 5).

### Elastic modulus and elongation at break

The elastic modulus and elongation at break were measured using the tensile test as presented in“Physical characterization of the developed PCL” and “Physical characterization of the commercially available masks”. The stress–strain curves on the tensile test of 4b45/2b20 and the commercially available mask (Type-S) at 60 °C are shown in Supplementary figure S2. The elastic muduli at 60 °C were 1.1 ± 0.3 MPa (4b45/2b20) and 46.3 ± 5.4 MPa (Type-S), and the elongation at break were 275.2% ± 25% (4b45/2b20) and 216 ± 15.2% (Type-S). The elastic moduli at 23 °C were 253.7 ± 24.5 MPa (4b45/2b20) and 282.0 ± 44.3 MPa (Type-S). Considerable differences were observed in the elastic modulus at 60 °C (*p* = 0.0357) and elongation at break (*p* = 0.0347), but no significant difference was found in the elastic modulus at 23 °C (*p* = 0.4) (Table 1).

**Table 1:** Elastic modulus and elongation at break of the 4b45/2b20 and the commercially available mask (Type-S).

Type of PCL	Elastic modulus (MPa)		Elongation at break at heating (%)
	60 °C	23 °C	60 °C
4b45/2b20	1.1 ± 0.3	253.7 ± 24.5	275.2 ± 25.0
Type-S	46.3 ± 5.4	282.0 ± 44.3	216.0 ± 15.2
*p*-value	0.0357	0.4	0.0347

PCL; poly-ε-caprolactone.

## Discussion

The melting point of 2b20 (39.7 °C) was lower than that for 4b45 (53.0 °C). The tendency for the melting point to decrease with the decrease in the molecular weight of PCL was the same as described in previous studies [^13,14,33^]. In contrast, the melting point of the newly developed PCL (4b45/2b20) was 46.0 °C, which was an intermediate point between 2b20 and 4b45 and was approximately 10 °C lower than the three commercially available masks (55.7 °C–56.5 °C). When the skin temperature is increased to approximately 50 °C, pain is gradually elicited, with grade 1 burns occurring within 3 min and grade 2 burns occurring within 6 min^32,33^. The PCL melting point of ≤ 50 °C is useful for decreasing the patient burden because the heating temperature can be < 50 °C which is less than that required for a grade 1 burn as just described. Crystallization temperature indicates the temperature around which the material begins to crystallize when creating the mask. Because of the time required for creating the mask when the crystallization temperature is far below room temperature, a crystallization temperature that is around room temperature is preferable for PCL. Therefore, the results shown herein for 4b45/2b20 with a melting point of 46 °C and crystallization temperature of 22.1 °C are useful and within a reasonable range for clinical use.

The elastic modulus of PCL during heating indicates the maximum pressure exerted on the patient’s face when creating the mask. The elastic modulus of 4b45/2b20 was significantly lower than that of the commercially available masks under same conditions. Additionally, the elongation at break indicates the amount of maximum elongation of the material at 60 °C, and that of 4b45/2b20 were significantly larger than the commercially available masks. Linear PCL made of tetramethylene glycol has lower transition temperature (melting point) than tetra-branched PCL and exhibits relatively large deformation ability. As tetramethylene glycol was used for the low melting point PCL of 4b45/2b20, its deformation ability was maintained despite the low melting point. The elastic modulus at 23 °C indicates the strength of the material at room temperature, and no significant differences between 4b45/2b20 and the three commercially available masks were observed (Table 1).

Based on these results, it was confirmed that the 4b45/2b20 has both a low melting point and a large deformation ability as well as clinically acceptable strength. The developed PCLs have an advantage because the handling method is the same as that for commercially available masks, and thus, it can be easily introduced into clinical settings. Additionally, in our previous research, we confirmed using computed tomography images and the similarity index that the PCL created in the same process as this research (“Development of the cross-linked PCL” and “Physical characterization of the developed PCL”) has almost a perfect shape memory performance^34^. Therefore, even when the mask creation fails for whatever reason (e.g., patient’s movement during mask formation), the shape can be restored to its original shape (i.e., flat) by heating the material again so that the mask can be easily recreated (Supplementary figure S3). Additionally, because the PCL has been approved for cleaning with 70% alcohol based on the recommended cleaning procedures according to the World Health Organization^35,36^, it can be used safely for at least six months to one year. Thus, the new PCL is promising as a material for a radiotherapy mask that can reduce the burden on patients.

This study has two major limitations. First, the tensile test of the commercially available masks could not be performed in actual conditions for clinical use, which is to heat for few minutes at 75 °C as recommended in the package insert and technical paper^9^. However, in the tensile test, to ensure uniform temperature of the material, it was necessary to heat the material for approximately 15 min. Because prolonged heating is not expected to be performed in the clinical setting, the commercially available masks are not sufficiently cross-linked, and the material melted during heating at 75 °C. Therefore, in this study, the tensile test at 75 °C could not be performed. To investigate the pressure to the patient’s body under clinical conditions, additional data is needed using a pressure sensor^37^. Second, this study did not evaluate any mixture other than 2b20 and 4b45. If the melting point is set too low, unintended deformation may occur during transportation depending on the season. Additionally, if the crystallization temperature is too close to room temperature, the time required for the mask to harden increases. The PCL which was mixed with 2b20 and 4b45 at a ratio of 33.3% and 66.6%, has sufficient deformation ability, appropriate melting point, and crystallization temperature. Therefore, the 4b45/2b20 is acceptable for clinical use. However, because the physical characteristics change based on the molecular weight, number of branches, and mixing ratio^27–30^, determining the optimal combination by conducting additional evaluations is necessary. In the future, using any reinforcement material also needs to be considered as regards good face-mask adhesion. Although issues, such as price and radiation scattering remain, it is necessary to evaluate by adding additives, such as cellulose to develop better materials.

## Conclusions

In this study, we developed a new PCL (4b45/2b20) by mixing two different PCLs and investigated the results of the analysis performed using DSC and tensile tests between the developed PCL and three commercially available masks. The new PCL 4b45/2b20 has a low melting point (46 °C) and large deformation ability, and its crystallization temperature and strength are within the clinically acceptable standards. These results show that 4b45/2b20 will be useful as a material for a thermoset mask for radiotherapy with reduced patient burden.

## Supplementary Information

Supplementary Information.

## Data Availability

The datasets during and/or analyzed during the current study available from the corresponding author on reasonable request.
