# Supplementary material for: Development of a new poly-ε-caprolactone with low melting point for creating a thermoset mask used in radiation therapy
Source: Sci Rep. 2021 Oct 14;11:20409. doi: 10.1038/s41598-021-00005-2 (PMC8516973; doi:10.1038/s41598-021-00005-2)
Supplement: Supplementary file 1 — Supplementary Information. [file 41598_2021_5_MOESM1_ESM.docx]

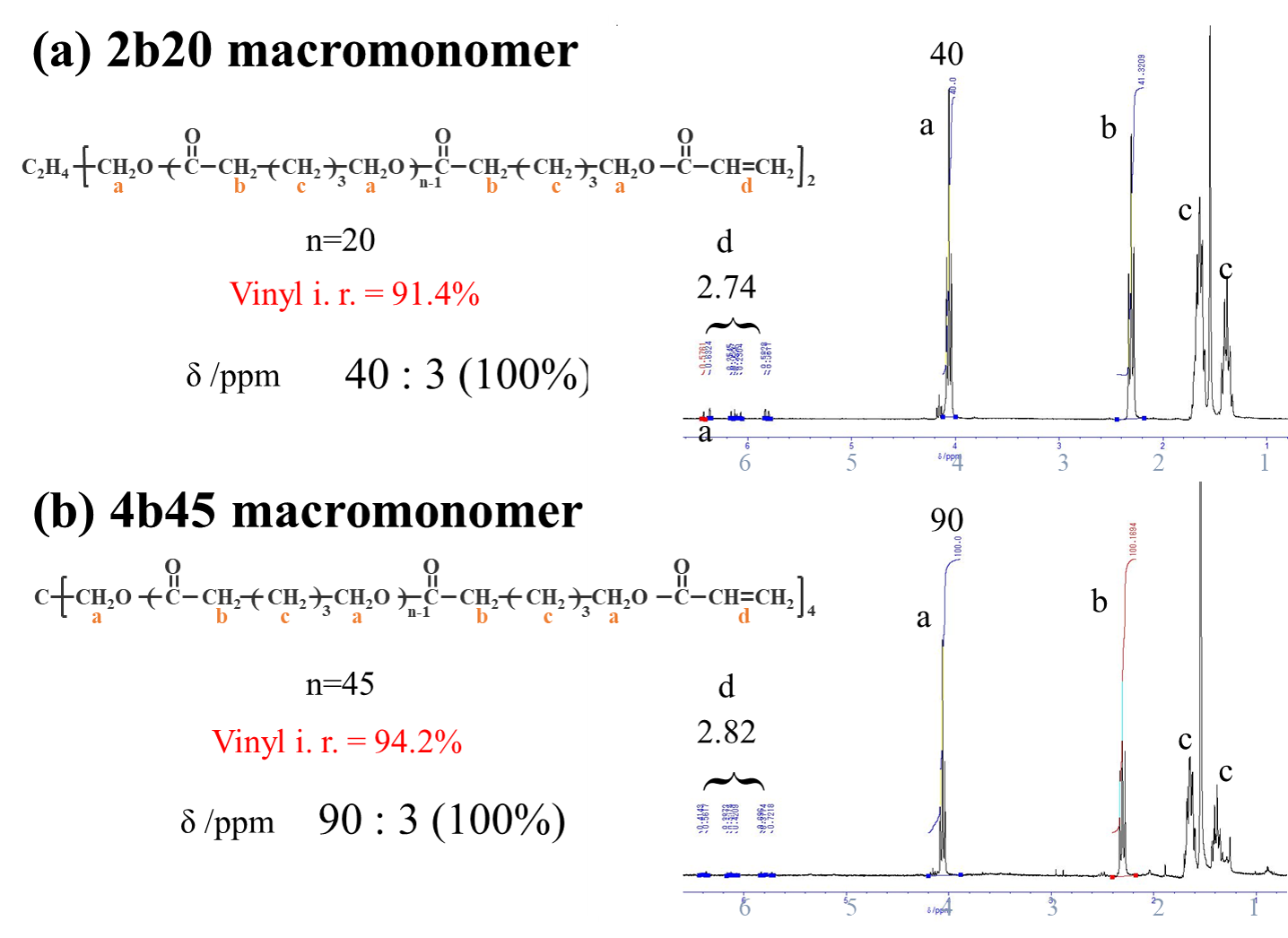


**Supplementary figure S1.** ^1^H NMR spectra of 2b20 macromonomer (a) and 4b45 macromonomer (b) PCL.　The macromonomers with high incorporation rates of 91.4% and 94.2% were obtained for 2b20 macromonomer and 4b45 macromonomer, respectively.


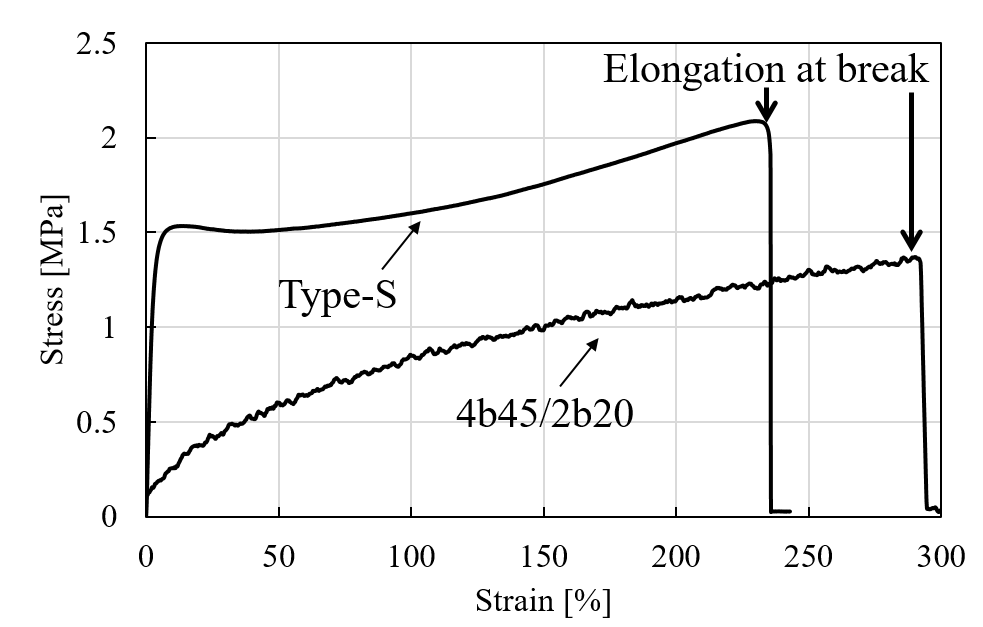


**Supplementary figure S2.** The stress–strain curve in the tensile test of 4b45/2b20 and the commercially available mask (Type-S) at 60°C.


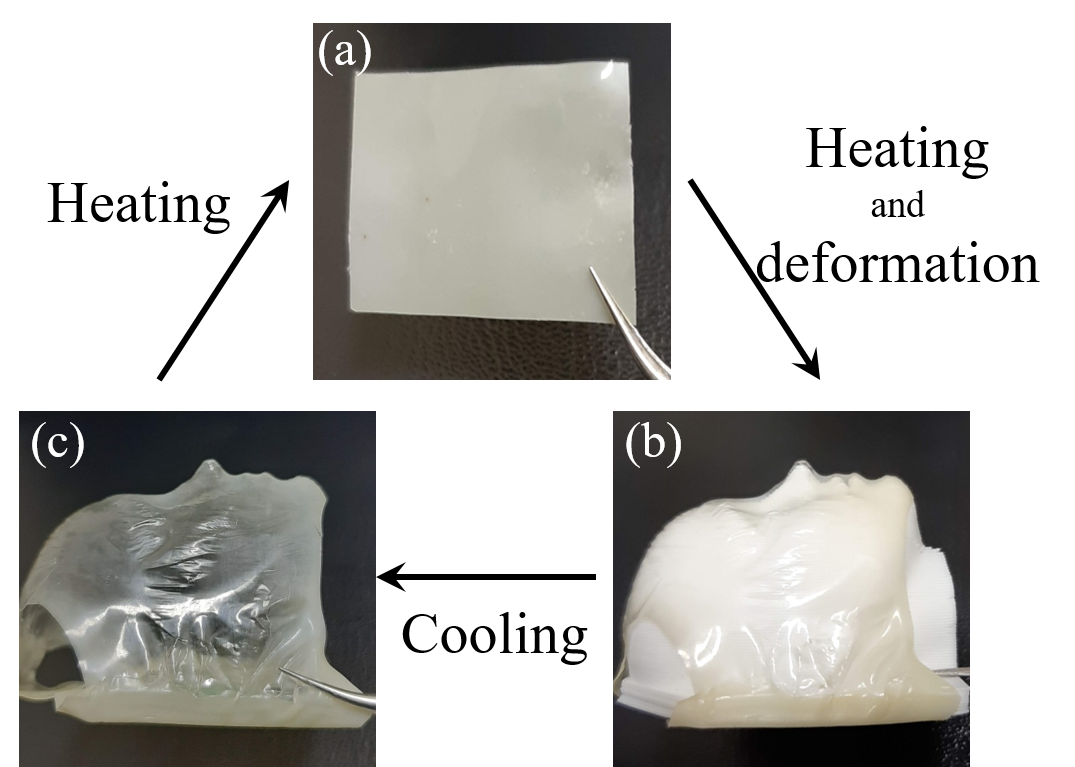


**Supplementary figure S3.** Photographs of the developed cross-linked poly-ε-caprolactone (PCL) (a) and demonstration of creating mask: (b) deformation after heating, and (c) cooling after deformation and the shape restored to that shown in (a) by reheating.
